# MiRNA-329 targeting E2F1 inhibits cell proliferation in glioma cells 

**DOI:** 10.1186/1479-5876-11-172

**Published:** 2013-07-17

**Authors:** Bingxiang Xiao, Li Tan, Benfu He, Zhiliang Liu, Ruxiang Xu

**Affiliations:** 1 The neurosurgery Department, General Hospital of Beijing Military Command of People's Liberation Army (PLA), Bei jing 100700, P. R. China; 2 The neurosurgery Department, Wuhan General Hospital, Guangzhou Command, PLA, Wu han 430070, P. R. China; 3 Center of Oncology and Hematology, the First Affiliated Hospital of Guangzhou Medical University, Guangzhou 510230, P. R. China; 4 Oncology Department, PLA 421 Hospital, Guangzhou 510318, P. R. China

**Keywords:** Glioma, E2F1, MiR-329, Cell proliferation

## Abstract

**Background:**

MicroRNAs have recently emerged as key regulators of cancers, miR-329 located on 14q32.31 is one of down-regulated miRNAs in glioma, but the function and molecular mechanisms of miR-329 in determining the malignant phenotype of human glioma are elusive. This study therefore was conducted to investigate the role of miR-329 in biological behaviors of human glioma LN18 and T98G cell lines and its molecular mechanisms.

**Methods:**

Nine patients with GBM were analyzed for the expression of miR-329 by quantitative RT–PCR. MiR-329 overexpression was established by transfecting miR-329 precursor into LN18 and T98G cells, and its effects on cell proliferation were studied using MTT assay, anchorage-independent growth ability assay, colony formation assays, Bromodeoxyuridine labeling and immunofluorescence.

The effects of miR-329 on cell cycle were studied by flow cytometry. The target of miR-329 was determined by luciferase assays. The regulation of miR-329 on Akt pathway was determined by western blot.

**Results:**

The E2F1 was identified as the target of miR-329. Overexpression of miR-329 blocked G1/S transition in LN18 and T98G cell lines, dramatically suppressed cell proliferation and the ability of colony formation. MiR-329 significantly decreased the phosphorylation levels of intracellular kinases Akt and expression of cyclin D1, but the expression of p**21** was upregulated, cell growth was suppressed by inhibiting E2F1-mediated Akt pathway.

**Conclusions:**

MiR-329 may inhibit cell proliferation in human glioma cells through regulating E2F1-mediated suppression of Akt pathway.

## Background

Glioma multiforme (GBM) is one of the most malignant brain tumors, with a median survival of ∼ 14 months [1]. Many of its variants demonstrate striking resistance to even aggressive treatment regimens. Recent advances have implicated a defined set of oncogenic pathways in the underlying biology of this tumor group [2]. Among these crucial signaling networks, the Akt pathway and E2F1 have emerged as being particularly important in glioma pathogenesis, which is correlated with poor prognosis in multiple glioma subtypes [3,4].

MicroRNAs (miRNAs) are a class of short, endogenous, non-coding RNA molecules that bind with imperfect complementarity to the 3′-untranslated regions (3′-UTRs) of target mRNAs, causing translational repression or message RNA degradation [5]. Recent studies have shown the importance of miRNAs in the normal regulation of gene expression during development and cell proliferation [6]. MiRNAs have also been shown to have critical roles in tumor biology [7-9], thus we may establish them as a relatively new and important class of oncogenes and tumor suppressor genes [10]. Aberrant expression of these miRNAs has been implicated in tumor growth and carcinogenesis.

MiR-329 is located on 14q32.31. The miRNA expressing profile of glioma samples and cell lines suggested that miR-329 is one of down-regulated miRNAs [11]. However, the function and molecular mechanism of miR-329 in determining the malignant phenotype of human glioma are elusive. In this study, we aimed to determine the role of miR-329 in determining the proliferation of glioma cells and study the regulatory mechanism of miR-329 in glioma cells. We constructed cell models of over-expressing miR-329 and down-expressing miR-329 in glioma cells and screened expressing levels of miR-329 and E2F1 in a group of glioma cells. E2F1 was identified as a significant target of miR-329 by luciferase assays, miR-329 was able to induce the G1/S arrest and inhibit proliferation of glioma cells through E2F1-mediated suppression of Akt pathway. So miR-329 may act as the role of tumor suppressor in glioma cells.

## Methods

### Ethics statement

For the use of clinical materials for research purposes, prior patients’ consent and approval were obtained from the General Hospital of Beijing Military Command of PLA.

### Clinical specimens

Glioma tissues were obtained from therapeutic procedures performed as routine clinical management at our institution. Tissue samples were resected during surgery and immediately frozen in liquid nitrogen for subsequent total RNA extraction. A total of 9 glioma (GBM) and 3 nonneoplastic brain specimens were included in our study.

### Cell Culture

Glioma cell lines, including A172, LN340, U118MG, LN464, SNB19, LN18, T98G, and U251MG were grown in DMEM medium (Invitrogen, Carlsbad, CA,USA) supplemented with 10% fetal bovine serum (Invitrogen, Carlsbad, CA,USA) and 1% penicillin/streptomycin. Cells were maintained in a humidified atmosphere at 37°C with 5% CO_2_.

### Construction of the 3′-UTR-luciferase plasmid and reporter assays

The E2F1 3′-UTR target site was amplified by PCR using the primers Fwd-5′-CATACTAGTTTCCAGAGATGCTCACCTTGT-3′ and Rev-5′- CTTAAGCTTAAGACAGAAGTGCTCTCACCGTC-3′ and cloned downstream of the luciferase gene in the pGL3-Report luciferase vector (Ambion, Austin, TX, USA). This vector was sequenced and named pGL3-E2F1-3′UTR. Reporter assay was performed at 48 h after transfection using the BriteLite plus reporter gene assay system (Perkin Elmer, Shelton, CT, USA).

### Reagents, antibodies and expression constructs

The candidate pre-miRNA-329 of double-stranded oligonucleotides was generated for cloning into the pcDNA6.2-GW/ EmGFP vector (Invitrogen, Carlsbad, CA, USA). The plasmid was sequenced and named pcDNA6.2-GW/EmGFP/ miR-329 (pre-miR-329). pcDNA6.2-GW/±EmGFP-miR-neg control plasmid (negative control miR) contained an insert that could be processed into mature miRNA but not to target any known vertebrate gene. The anti-miR molecules were purchased from Ambion (Austin, TX, USA). Full-length E2F1 expression vector in the mammalian expression vector, pCMV-SPORT6, was purchased from Open Biosystems (Huntsville, AL, USA). The control plasmid, pCMVSPORT6, was generated by excising the E2F1 insert through restriction digestion. Antibodies specific for Akt, phospho-AktSer473, p21 and cyclin D1 were purchased from Cell Signaling Technology (Beverly, MA, USA). The anti-E2F1 antibody was purchased from Santa Cruz Biotechnology (Santa Cruz, CA, USA). Akt inhibitor IV was purchased from Calbiochem (EMD Chemicals Inc, San Diego, CA, USA). SiE2F1#1 and SiE2F1#2 were from invitrogen (Invitrogen, Carlsbad, CA, USA). The vectors pBABE-E2F1 overexpressing E2F1 and pBABE-E2F1-3′UTR including miR-329 3′UTR binding site were constructed.

### Quantitative RT–PCR assays for mature miRNA

The reverse transcription reactions of cell lines or human glioma specimens were performed in a reaction containing 50 ng small RNA. Amplification and detection of specific products were performed with the Roche LightCycler detection system with the cycle profile according to the mirVana qRT–PCR miRNA Detection Kit (Ambion, Austin, TX, USA ). The relative gene expression was calculated by comparing the cycle times for each target PCR. The target PCR Ct values were normalized by subtracting the internal control of 5S rRNA Ct value.

### 3-(4, 5-Dimethyl-2-thiazolyl)-2, 5-diphenyl-2H-tetrazolium bromide (MTT) assay

Cells, seeded on 96-well plates, were stained at indicated time point with 100 μL sterile MTT dye (0.5 mg/ml, Sigma, St. Louis, MO, USA) for 4 h at 37°C, followed by removal of the culture medium and addition of 150 μL of dimethyl sulphoxide (DMSO) (Sigma, St. Louis, MO, USA). The absorbance was measured at 570 nm, with 655 nm as the reference wavelength. All experiments were performed in triplicates.

### Anchorage-independent growth ability assay

Five hundred cells were trypsinized and suspended in 2 ml complete medium plus 0.3% agar (Sigma, St Louis, MO, USA). The agar–cell mixture was plated on top of a bottom layer with 1% complete medium agar mixture. After 10 days, viable colonies that contained more than 50 cells or were larger than 0.1 mm were counted. Colony size was measured with an ocular micrometer and colonies greater than 0.1 mm in diameter were counted. The experiment was performed for three independently times for each cell line.

### Colony formation assays

Cells were plated on 6-well plates (0.5 × 10^3^ cells per plate) and cultured for 10 days. The colonies were stained with 1.0% crystal violet for 30s after fixation with 10% formaldehyde for 5 min.

### Bromodeoxyuridine labeling and immunofluorescence

Cells grown on coverslips (Fisher, Houston, TX, USA) were incubated with bromodeoxyuridine (BrdUrd) for 1 h and stained with anti-BrdUrd antibody (Upstate, Temecula, CA, USA) according to the manufacturer’s instruction. Gray level images were acquired under a laser scanning microscope (Axioskop 2 plus, Carl Zeiss Co. Ltd., Jena, Germany).

### Luciferase assays

Cells (4×10^4^) were seeded in triplicates in 24-well plates and allowed to settle for 24 h. The miR-329 mimics, the miR-329-mut, and anti-miR-329 inhibitor purchased from RiboBio (RiboBio Co.Ltd, Guangzhou, Guangdong) were respectively transfected into glioma cells using the Lipofectamine 2000 reagent (Invitrogen, Carlsbad, CA, USA) with 100 ng of pGL3-E2F1-3′UTR, plus 10 ng of pRL-TK renilla plasmid (Promega, Madison, WI, USA) according to the manufacturer’s recommendation. Luciferase and renilla signals were measured 48 h after transfection using the Dual Luciferase Reporter Assay Kit (Promega, Madison, WI, USA) according to a protocol provided by the manufacturer. Three independent experiments were performed and the data are presented as the mean + SD.

### Flow cytometry analysis

All cells in a culture dish were harvested by trypsinization, washed in ice-cold PBS, and fixed in 80% ice-cold ethanol in PBS. Before staining, the cells were spun down in a cooled centrifuge and resuspended in the cold. Bovine pancreatic RNAase (Sigma, St. Louis, MO, USA) was added at a final concentration of 2 mg/mL, and cells were incubated at 37°C for 30 min, followed by incubation in 20 mg/mL of propidium iodide (Sigma, St. Louis, MO, USA) for 20 min at room temperature. 50,000 cells were analyzed on a flow cytometer (FACSCalibur; BD Biosciences).

### Statistical analysis

The Student’s t -test was used to evaluate the significant difference of two groups of data in all the pertinent experiments. A *P* value < 0.05 (using a two-tailed paired t test) was thought to be significantly different for two groups of data.

## Results

### Downregulation of miR-329 in glioma

**Figure 1:**
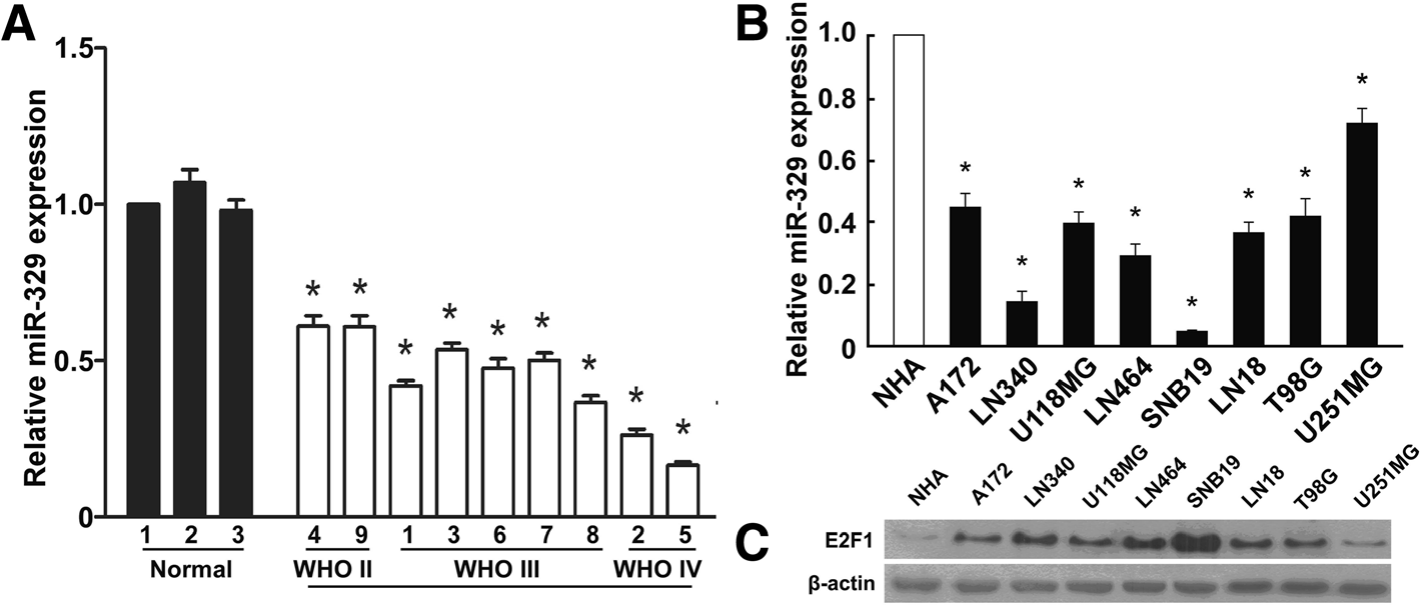
**Expression analysis of miR-329 in glioma cell lines and glioma tissues. A)** Real-time PCR analysis of miR-329 expression in three nonneoplastic brain specimens and primary glioma tissues of nine individual patients. **B)** Real-time PCR analysis of miR-329 expression in primary normal human astrocytes (NHA) and glioma cell lines (including A172, LN340, U118MG, LN464, SNB19, LN18, T98G, and U251MG). The average miR-329 expression was normalized by U6 expression. Each bar represents the mean of three independent experiments. * *P* < 0.05. **C)** Western blotting analysis of E2F1 in primary normal human astrocytes (NHA) and glioma cell lines (including A172, LN340, U118MG, LN464, SNB19, LN18, T98G, and U251MG), β-actin served as the loading control.

First, we examined miR-329 expression in GBM cell lines. Real-time RT-PCR was performed on a panel of 8 human GBM cell lines and primary normal human astrocytes. MiR-329 expression of each cell line was compared to the average expression level of primary normal human astrocytes (NHA). As shown in Figure 1B, miR-329 expression levels of all cell lines were lower than that of NHA, while expression levels of E2F1 in the cell lines were higher (Figure 1C). Downregulation of miR-329 was also found in clinical samples compared with nonneoplastic brain specimens (Figure 1A).

### MiR-329 overexpression reduces cell proliferation in glioma

**Figure 2:**
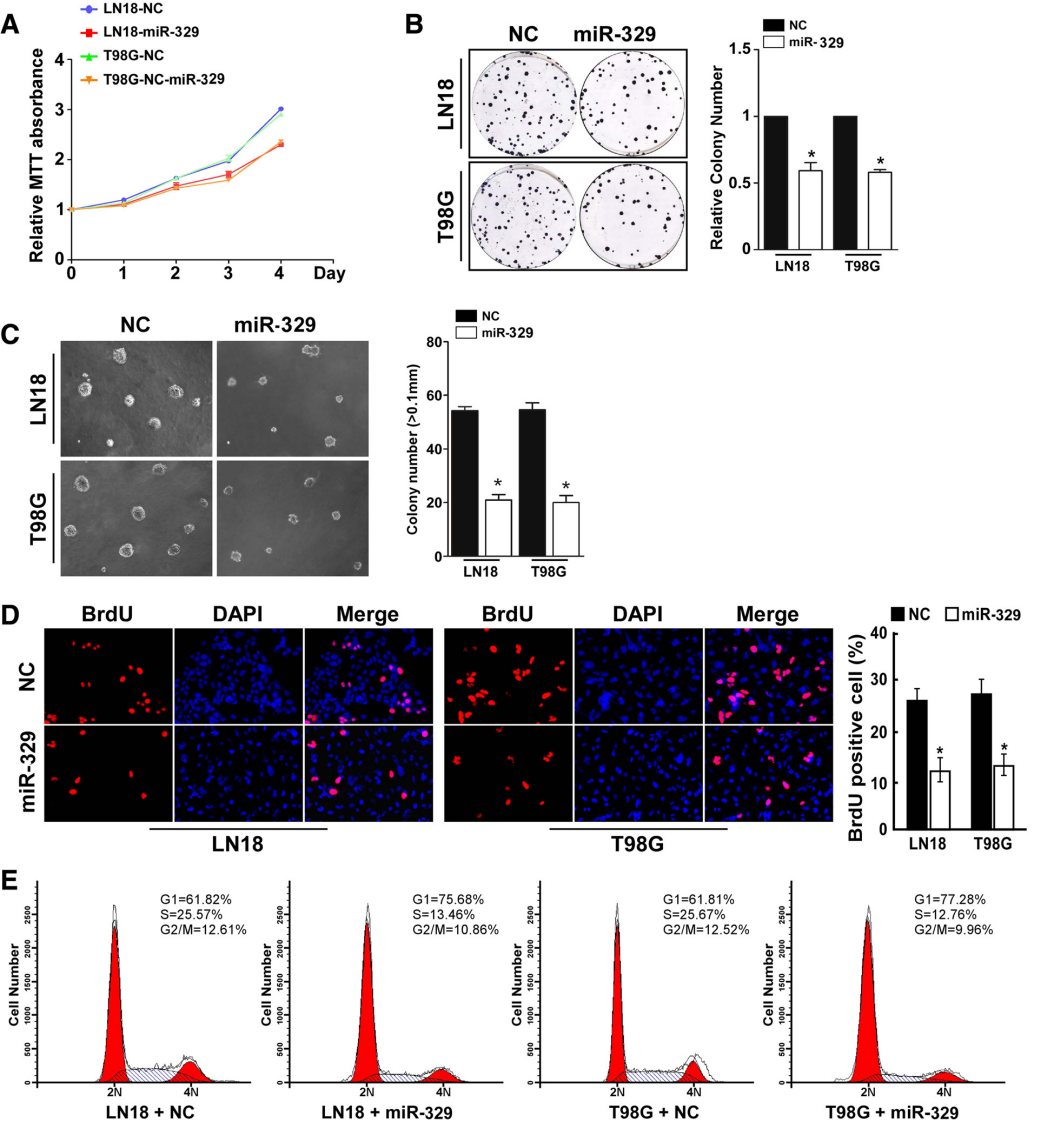
**Upregulation of miR-329 inhibits the cell proliferation in glioma. A)** MTT assays revealed that upregulation of miR-329 inhibited cell growth of glioma cell lines of T98G and LN18. **B)** Representative micrographs (left) and quantification (right) of crystal violet stained cell colonies. **C)** Upregulation of miR-329 inhibited glioma cell tumorigenicity as determined by the anchorage-independent growth assay. Representative micrographs (up) and quantification of colonies that were larger than 0.1 mm (down) were scored. **D)** Representative micrographs (left) and quantification of BrdU incorporating-cells after transfection with miR-329 or NC. Each bar represents the mean of three independent experiments. **E)** Flow cytometric analysis of the indicated glioma cells transfected with NC or miR-329. Each bar represents the mean of three independent experiments. * *P* < 0.05.

To explore the role of miR-329 downregulation in the development and progression of glioma, we examined its effect on cell proliferation. A MTT assay showed that miR-329 upregulation significantly inhibited the proliferation rate of LN18 and T98G glioma cells (Figure 2A), and this was further confirmed by a colony formation assay (Figure 2B). Strikingly, we found that enforced expression of miR-329 in LN18 and T98G glioma cells drastically inhibited their anchorage-independent growth ability (Figure 2C), as shown by decreased colony numbers and sizes, these results suggested that miR-329 upregulation inhibits glioma cell tumorigenicity in vitro. Using a BrdU incorporation assay, we found that the percentage of cells in S phase was dramatically decreased in miR-329-overexpressing LN18 (12.25%) and T98G (13.43%) cells compared with control cells (LN18 cells, 27.25%; T98G cells, 28.43%; Figure 2D). Similarly, the result of flow cytometry showed that miR-329 overexpression decreased the percentage of cells in S phase and significantly increased the percentage of cells in G1/G0 (Figure 2E). Collectively, our results suggest that miR-329 may induce the G1/S arrest and inhibit cell proliferation of glioma.

### MiR-329 inhibition increases cell proliferation in glioma

**Figure 3:**
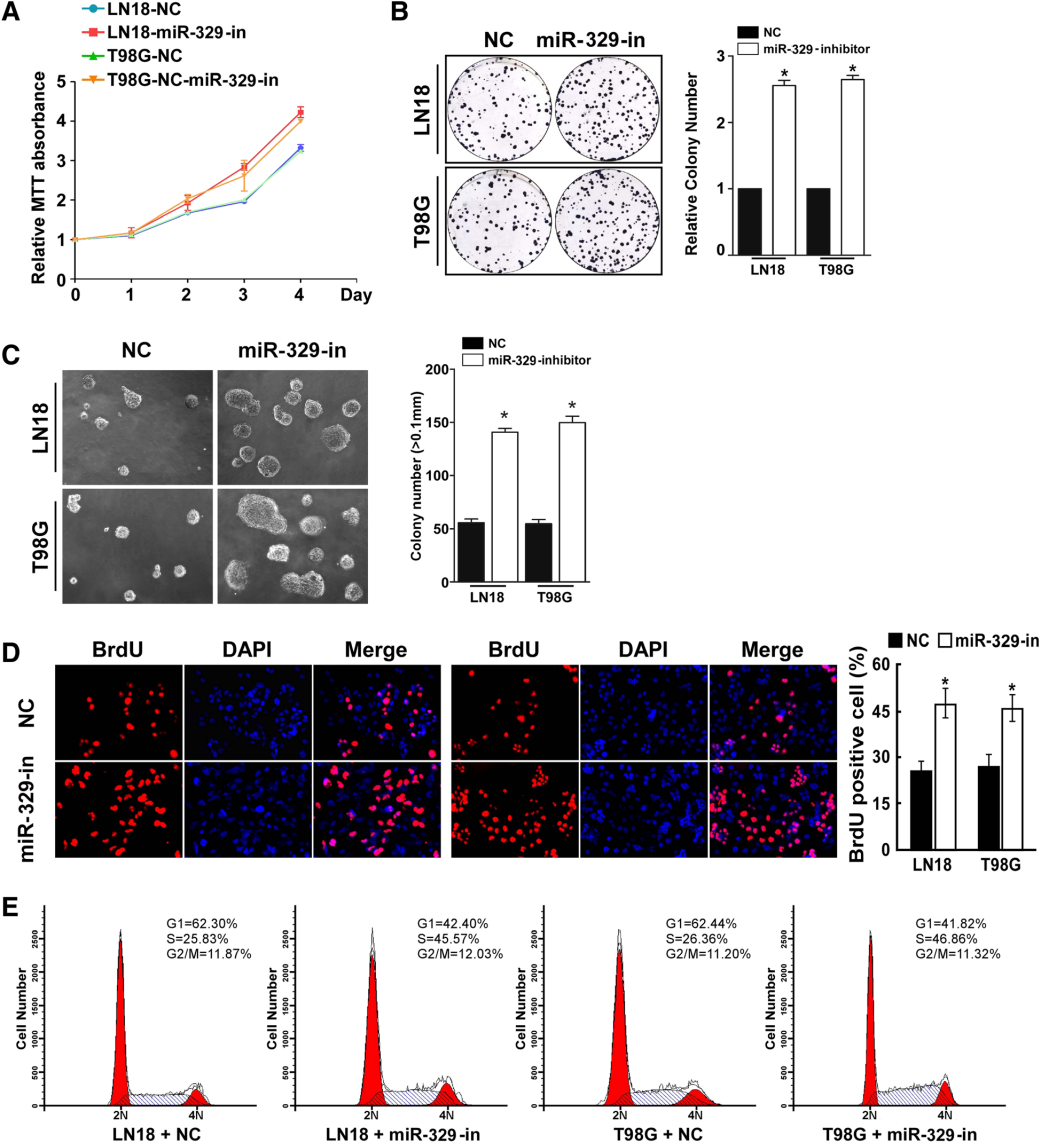
**Inhibition of miR-329 promotes cell proliferation in glioma. A)** MTT assays revealed that inhibition of miR-329 increased cell growth of glioma cell lines of T98G and LN18. **B)** Representative micrographs (left) and quantification (right) of crystal violet stained cell colonies. **C)** Inhibition of miR-329 increased the anchorage-independent growth of glioma cells. Representative micrographs (up) and quantification of colonies that were larger than 0.1 mm (down) were scored. **D)** Representative micrographs (left) and quantification of BrdU incorporating-cells after transfection with miR-329 inhibitor or NC. **E)** Flow cytometric analysis of the indicated glioma cells transfected with NC or miR-329 inhibitor. Each bar represents the mean of three independent experiments. * *P* < 0.05.

We further examined the effect of miR-329 inhibition on cell proliferation in glioma. Consistent with above mentioned results, MTT and colony formation assays showed that miR-329 suppression dramatically increased the growth rate of both LN18 and T98G glioma cells as compared with that of control cells transfected with negative control (NC) (Figures 3A and 3B). In addition, the anchorage-independent growth ability of LN18 and T98G glioma cells was significantly increased in response to miR-329 inhibitor (Figure 3C). Furthermore, we found that transfection of the miR-329 inhibitor drastically increased the percentage of cells in the S peak but decreased the percentage of cells in the G0/G1 peak (Figures 3D and 3E).These results suggested that the proliferative effect of inhibiting miR-329 in glioma cells may occur through regulation of G1/S transition.

### MiR-329 directly targets E2F1 in glioma cells

**Figure 4:**
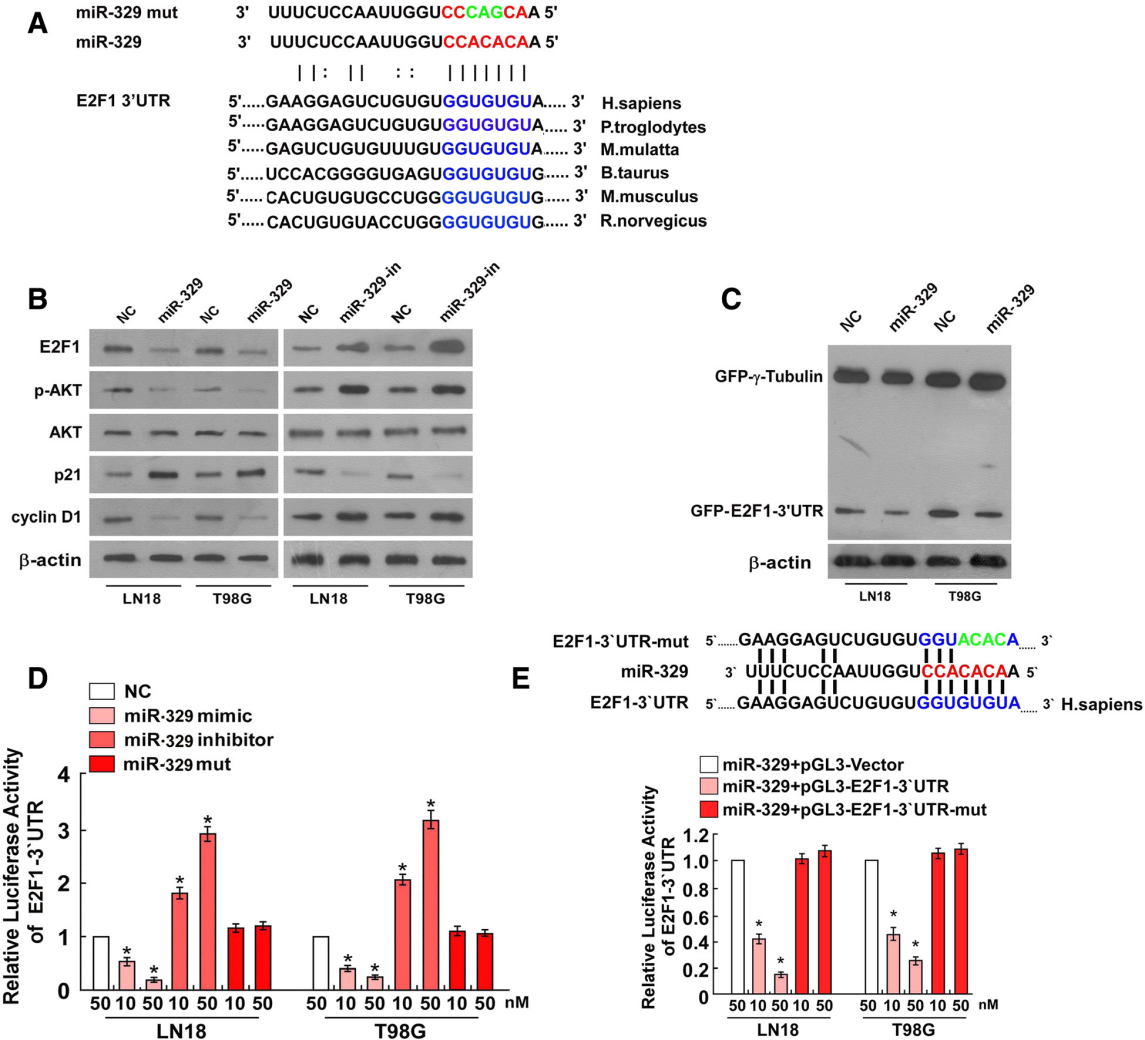
**MiR-329 suppresses E2F1 expression and Akt pathway. A)** Predicted miR-329 target sequence (blue) in the 3′UTR of E2F1 (E2F1-3′UTR) and positions of the mutated nucleotides (green) in the miR-329 (miR-329 mut). **B)** Western blotting analysis of E2F1,pAkt, Akt, p21, cyclinD1 expression in cells transfected with miR-329 or the miR-329 inhibitor, β-actin served as the loading control. **C)** Western blotting analysis of GFP expression in the indicated cells, β-actin served as the loading control. **D)** Luciferase reporter assay of the indicated cells transfected with the pGL3-E2F1-3′UTR reporter and increasing amounts (10, 50 nM) of miR-329 mimic, miR-329 inhibitor or miR-329 mut oligonucleotides. Bars represent the mean ± SD of three independent experiments. * *P* <0.05. **E)** Luciferase reporter assay of the indicated cells transfected with pGL3-E2F1-3′UTR or pGL3-E2F1-3′UTR-mut reporter with increasing amounts (10, 50 nM) of miR-329 mimic. * *P* <0.05.

**Figure 5:**
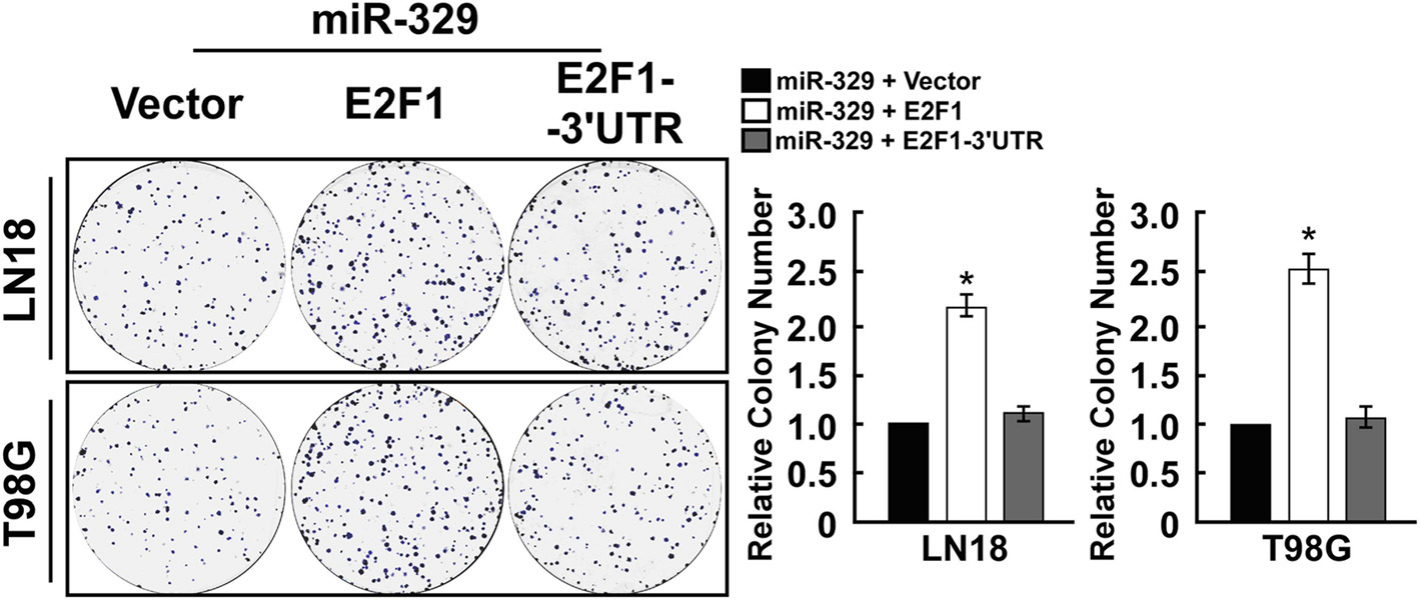
**MiR-329 inhibits cell proliferation through targeting E2F1 3′UTR in glioma. A)** Representative micrographs (left) and quantification (right) of crystal violet stained cell colonies.

Analysis with the use of two publicly available algorithms (TargetScan and miRanda), we found that E2F1 mRNA is theoretically the target gene of miR-329 (Figure 4A). Importantly, western blotting analysis showed that ectopic expression of miR-329 dramatically decreased, but inhibition of miR-329 increased E2F1 protein expression in both LN18 and T98G glioma cells (Figure 4B). The pBABE-E2F1 overexpressing E2F1and pBABE-E2F1-3′UTR were respectively transfected into glioma cells with miR-329 mimic expressing using the Lipofectamine 2000 reagent. The result of colony formation assay showed overexpressing E2F1 significantly increased the proliferation rate of LN18 and T98G glioma cells compared with that cells expressing E2F1-3′UTR (Figure 5A), the rescuing experiment further confirmed that the inhibitory role of miR-329 in glioma cells may be mediated by E2F1. To examine whether miR-329 downregulation of E2F1 was mediated by the 3′-untranslated region (3′UTR) of E2F1, we subcloned the E2F1 3′UTR fragment, containing the miR-329 binding site, into pEGFP-C1 and pGL3 dual luciferase reporter vectors. As shown in Figure 4C, overexpressing miR-329 only decreased expression of a GFP vector containing the E2F1 3′UTR, but had no effect on GFP-γ-tubulin expression, the result suggested that miR-329 specifically affected the 3′UTR of E2F1. To validate that miR-329 can directly bind to and regulate the levels of E2F1 mRNA through the predicted binding sites, a mutant version of the reporter (pGL3–E2F1-3′UTR-mut plasmids) and altering bases in the putative miR-329 binding sites (miR-329 mut) were used in luciferase reporter assay. The consistent and dose-dependent reduction of luciferase activity was observed following miR-329 transfection in both glioma cells, the reporter assay revealed that the repressive effect of miR-329 on the luciferase activity of E2F1 3′UTR was abolished by miR-329 inhibitor but did not have the effect in the miR-329 mut group (Figure 4D). The overexpression of miR-329 also efficiently reduced the expression of the luciferase reporter in the pGL3–E2F1-3′UTR group but did not have the effect in the pGL3–E2F1-3′UTR-mut group (Figure 4E). Collectively, these results demonstrate that E2F1 is a bona fide target of miR-329.

### MiR-329 inhibites the Akt pathway

Several studies have shown the importance of the Akt kinase and mitogen-activated protein kinase (MAPK) signaling pathways in regulating cell growth, survival and apoptosis. Such as Akt, p21 and cyclin D1, which were important in signal transduction and regulating cell cycle. Consistent with above mentioned results, miR-329 is found to significantly decrease the phosphorylation levels of intracellular kinases Akt, and upregulate the expression of p**21** in miR-329-overexpressing cells, while p**Akt** phosphorylation was increased and the expression of p**21was inhibited** in the miR-329-inhibited cells. Interestingly, the protein level of cyclin D1, a CDK regulator important for regulating the G1/S transition, was downregulated in LN18 and T98G glioma cells transfected with miR-329 mimic, but increased in the cells transfected with miR-329 inhibitor, compared with control cells (Figure 4B).

**Figure 6:**
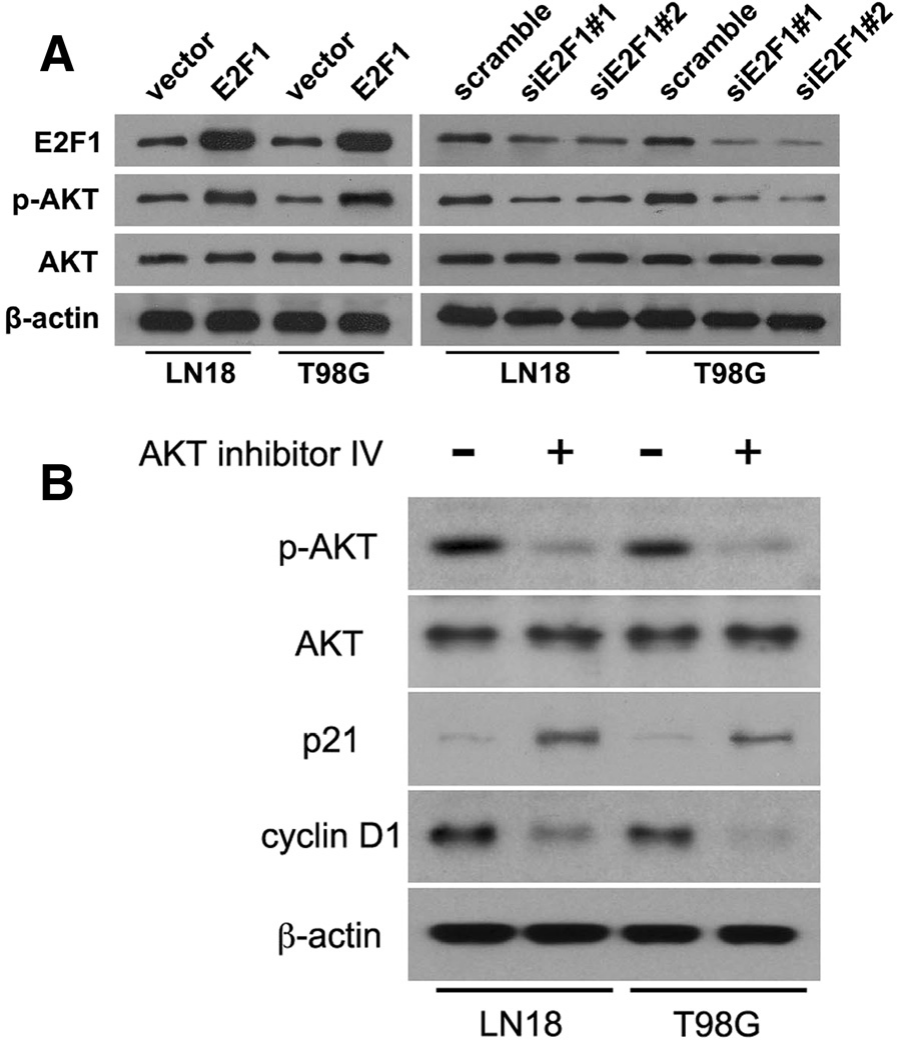
**E2F1 mediates suppression of Akt pathway. A)** Western blotting analysis of pAkt and Akt after alternation of E2F1 expression. **B)** Western blotting analysis of pAkt, Akt, p21, cyclinD1 expression after Akt inhibitor IV treatment, β-actin served as the loading control.

E2F1 overexpression in glioma cells can cause the phosphorylated level of Akt increase, interfering with the expression of E2F1 can decrease the phosphorylated level of Akt (Figure 6A). The levels of Akt phosphorylation are decreased by treatment with Akt inhibitor IV, in which the p**21** is significantly increased and cyclin D1 is downregulated (Figure 6B). These results provided further evidence that miR-329 may negatively regulate the Akt survival pathway through E2F1-mediated suppression of Akt phosphorylation and play an important role in cell proliferation of glioma.

## Discussion

The key finding of the current study is that miR-329 expression was markedly downregulated in glioma cells and glioma tissues, compared with that in nonneoplastic brain specimens and primary normal human astrocytes (NHA). Furthermore, ectopic expression of miR-329 inhibited the cell proliferation and anchorage-independent growth of glioma, while miR-329 inhibition had the opposite effect, this point was further confirmed in Additional file 1: Figure S1. Our results suggested that anti-proliferation of miR-329 may be related with theharrest of G1/S in glioma cells. This is the first study to show that the oncogene E2F1 is negatively regulated by miR-329 at the posttranscriptional level through a specific target site (nt 1655–1661) within the 3′-UTR. E2F1 was verified as a promising target gene, which is related with G1/S transition. We also showed that miR-329 inhibits proliferation through E2F1-mediated suppression of Akt phosphorylation in glioma cells.

E2F1 is a downstream regulator of the Rb pathway, which is capable of inducing cell proliferation and cell cycle progression by regulating mTORC1 activity [12-15]. The main molecular regulator of the G1 checkpoint is the p16/pRb/E2F pathway and abnormalities in every member of this pathway are present in most of gliomas. However, others have shown overexpression of E2F1 in gliomas triggered apoptosis and suppressed tumor growth in vitro and in vivo [16]. Regardless of p53 status, apoptosis induced by overexpression of E2F1 in glioma cell lines was further enhanced by treatment with ionizing radiation [17]. So the function of E2F1 seems to be paradoxical in glioma.

Recently, a cluster of miRNAs determining the regulation of E2F1 expression has been noticed. For example, miR-106b, miR-205, miR-15, miR-16, miR-17, miR-20a, and miR-34a which were located on separated miRNA clusters can cooperate to inhibit E2F1 translation [18-22]. It has been shown that the low expression of miR-106a in human glioma specimens is significantly correlated with high levels of E2F1 protein and high-grade glioma, E2F1 is a direct functional target of miR-106a, the suppressive effect of miR-106a on the glioma may result from inhibition of E2F1 via post-transcriptional regulation [23]. Expression of several members of miR-17-92 was also significantly increased with tumor grade progression. Mir-17-92 inhibition was associated with increased messenger RNA (mRNA) and/or protein expression of E2F1 [24].

Our results showed that the expression of E2F1 was regulated by miR-329 and the level of E2F1 protein expression was inversely correlated with miR-329 expression in glioma cells. E2F1 functions as an oncogene in gliomas, the oncogenic function of E2F1 may be mainly marked in glioma. [25]. The major effect of E2F1 has been shown to be mediated through the activation of the Akt-signaling pathway [26].

Akt, a pathway activated in the majority of GBMs, represents a nodal point in the signaling of malignant growth. PhosphoAkt expression levels were shown to be elevated in gliomas in vitro and in vivo [27,28]. Activated Akt phosphorylates many downstream proteins that can have a multitude of effects on a cell. Two of Akt’s downstream targets are major players in the regulation of cell cycle entry. GSK-3 promotes cell cycle entry by phosphorylating Cyclin D1-Cdk4 complexes, activated AKT phosphorylates GSK-3β to inactivate it [29,30]. This stabilized cyclin D1 will leads to the accumulation of Cyclin D1 in the cell [29,31]. Cyclin D1 is important for regulating the G1/S transition [32]. A second downstream target of Akt is MDM2 which is an inhibitor of p53 [33], so that Akt is free to block p53 activity causing self-sufficiency in growth signals and limitless replication potential. P21 is one of the downstream effectors p53 and play the important regulation at G1/S transition and repair damaged DNA [34-36]. Over activation of Akt pathway (along with several other mutated pathways) can be involved in the regulation of cell growth and help a normal astrocyte progress into a malignant glioma.

Our results showed that miR-329 significantly decrease the expression level of intracellular p-Akt and E2F1 in miR-329-overexpressing cells. The important downstream targets of Akt in the regulation at G1/S transition, cyclin D1 and p21 were respectively downregulated and upregulated in miR-329-overexpressing cells. Alternation of E2F1 may positively affect the expression level of p-Akt. Furthermore, we also examined whether the Akt inhibitor can synergize with miR-329 in inhibiting proliferation in glioma cells, the levels of Akt phosphorylation are decreased by treatment with Akt inhibitor IV, in which the p**21** is significantly increased and cyclin D1 is decreased. Overexpression of E2F1 was shown to be oncogenic and predisposing cells to neoplastic transformation [12-15]. However, the major effect of E2F1 in conferring numerous survival advantages has been shown to be mediated through the activation of the Akt-signaling pathway [26]. In this study, we showed that miR-329 can significantly decrease the phosphorylation of Akt, miR-329 might achieve anti-proliferation and induce G1/S transition through negatively regulating E2F1 expression and inhibiting Akt pathway at least in part. Qur analysis revealed that restoring miR-329 expression attenuated protein level of E2F1 by posttranscription regulation, and inhibited cell cycle progression in glioma. Targeting to the miR-329/E2F1 interaction or rescuing miR-329-expression may be a new therapeutic application to treat glioma patients in the future.

## Conclusions

We have examined the role of miR-329 in biological behaviors of human glioma cells and its molecular mechanism. MiR-329 might suppress the ability of colony formation and induce G1/S transition in glioma cells. Restoring miR-329 expression attenuated protein level of E2F1 by posttranscription regulation, E2F1 gene was identified as the target of miR-329. The anti-proliferation effect of miR-329 partly is related with the inhibition of Akt pathway mediated E2F1. However, the biological function of miR-329 in glioma was not be fully elucidated, the role of it in protection against apoptosis and in cell survival was still worth further studying. Therefore, miR-329 might be a potential therapeutic target for glioma that requires more in-depth analysis.

## Additional file

**MiR-329 inhibits cell proliferation in SNB19 and U251 glioma cells.** As shown in Figure 1B, miR-329 expression levels of SNB19 cell lines were lower than that of other cell lines while expression levels of it in the U251 cell lines were higher than that of other cell lines. E2F1 expression levels of SNB19 cell lines were higher than that of other cell lines while expression levels of it in the U251cell lines were lower (Figure 1C). The result of MTT showed that the growth speed of U251 is significant slower than that of SNB19 (Additional file 1: Figure S1A, S1B). Overexpression of miR-329 in SNB19 cells inhibited the proliferation ability of cells and the proliferating cells were significantly decreased, this was confirmed by colony formation assay and BrdU incorporation assay (Additional file 1: Figure S1C, S1D, S1E). Inhibition of the miR-329 expression in U251 increased the proliferation ability of cells and the proliferating cells were significantly increased, this was shown in colony formation assay and BrdU incorporation assay (Additional file 1: Figure S1C, S1D, S1E).

## Abbreviations

PLA: People's liberation army; GBM: Glioma multiforme; ECL: Enhanced chemiluminescence; FCM: Flow cytometry; miRNA: MicroRNA; mRNA: Messenger RNA; PI: Propidium iodide; SDS-PAGE: Sodium dodecyl sulfate polyacrylamide gel electrophoresis; UTR: Untranslated region. MTT, 3-(4,5-Dimethyl-2-thiazolyl)-2,5-diphenyl-2H-tetrazolium bromide; DMSO: Dimethyl sulphoxide; NC: Negative control.

## Competing interests

The authors declare that they have no competing interests.

## Authors’ contributions

Bingxiang Xiao identified and recruited patients and organised sample collection, performed quantitative RT–PCR. Li Tan and Benfu He performed western bolt, cell proliferation and Luciferase assays. Zhijun Yang and Zhiliang Liu performed cell cycle assay and construct of vectors. Ruxiang Xu participated in the project design, coordination the experiments, and manuscript preparation. All authors read and approved the final manuscript.

## Supplementary Material

Additional file 1: Figure S1 **MiR-329 inhibites cell proliferation in SNB19 and U251 glioma cells. A**) Western blotting analysis of E2F1 in primary normal human astrocytes (NHA) and glioma cell lines (including A172, LN340, U118MG, LN464, SNB19, LN18, T98G, and U251MG), β-actin served as the loading control. **B, C**) MTT assays revealed that the cell growth of glioma cell lines of SNB19 and U251. **D**) Quantification of crystal violet stained cell colonies were scored in glioma cell lines of SNB19 and U251. **E**) Quantification of BrdU incorporating-cells after transfection with miR-329, miR-329 inhibitor or NC. Each bar represents the mean of three independent experiments in glioma cell lines of SNB19 and U251. Click here for file
