# Supplementary figures and images for: MiRNA-329 targeting E2F1 inhibits cell proliferation in glioma cells
Source: J Transl Med. 2013 Jul 17;11:172. doi: 10.1186/1479-5876-11-172 (PMC3750231; doi:10.1186/1479-5876-11-172)

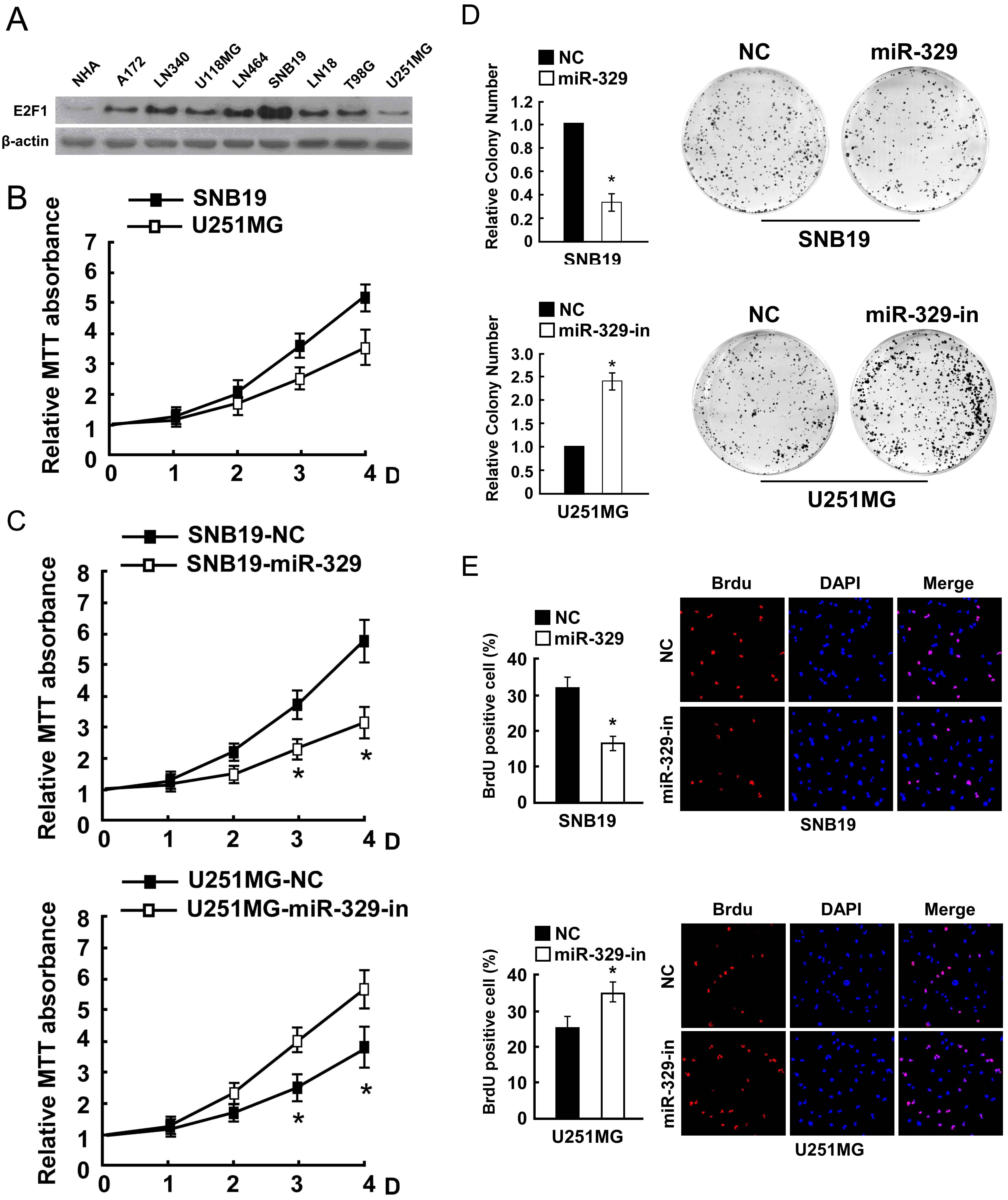

Supplement: Additional file 1: Figure S1 — MiR-329 inhibites cell proliferation in SNB19 and U251 glioma cells. A) Western blotting analysis of E2F1 in primary normal human astrocytes (NHA) and glioma cell lines (including A172, LN340, U118MG, LN464, SNB19, LN18, T98G, and U251MG), β-actin served as the loading control. B, C) MTT assays revealed that the cell growth of glioma cell lines of SNB19 and U251. D) Quantification of crystal violet stained cell colonies were scored in glioma cell lines of SNB19 and U251. E) Quantification of BrdU incorporating-cells after transfection with miR-329, miR-329 inhibitor or NC. Each bar represents the mean of three independent experiments in glioma cell lines of SNB19 and U251. [file 1479-5876-11-172-S1.jpeg]
